# Feasibility of patient-reported outcomes collection in clinical routine at a radiotherapy department

**DOI:** 10.2340/ao.v65.45546

**Published:** 2026-04-22

**Authors:** Ingrid Fagerström Kristensen, Anton Linnér, Martin P. Nilsson, Viktor Rogowski, Per Munck af Rosenschöld, Mats Jerkeman

**Affiliations:** a Oncology, Lund University, Lund, Sweden; b Radiation Physics, Department of Hematology, Oncology and Radiation Physics, Skåne University Hospital, Sweden, Lund, Sweden; c Oncology, Department of Hematology, Oncology and Radiation Physics, Skåne University Hospital, Skane University Hospital, Lund, Sweden; d Medical Radiation Physics, Lund University, Lund, Sweden

**Keywords:** Health-related quality of life, cancer, radiotherapy, patient-reported outcome measurement

## Abstract

**Background and purpose:**

Routine collection of patient-reported outcome measures (PROMs) may provide valuable real-world data on quality of life (QoL) and treatment tolerability, complementing clinical outcomes. This study evaluated the feasibility of launching a semi-automated, department-wide PROM collection program at a large tertiary radiotherapy centre.

**Patients and methods:**

Patients ≥ 18 years referred for radiotherapy were invited to participate. Exclusion criteria were inability to provide informed consent, severe cognitive conditions, or language barriers. PROMs were collected using a web-based application (“Blå Appen”) at baseline, end-of-treatment, and follow-up intervals, using the EORTC QLQ-C30 questionnaire (Version 3).

**Results:**

During the study period (January 2022 to April 2025), 12,472 patients underwent treatment in the department. A total of 4,764 provided informed consent for study participation, where 3,699 (77.6%) remained after exclusion due to data loss or data inconsistency. Among these, 3,056 patients (82.6%) provided at least one usable PROM questionnaire at baseline. Participants were younger (median age 69 years vs. 71 years). Overall, global health status was relatively high at baseline and remained stable or slightly improved at one-year follow-up. To further investigate clinically significant changes in QoL over time, we evaluated the proportion of patients in each diagnostic category who experienced a change of > 10 points in selected QLQ-C30 scales between baseline and follow-up. Breast cancer patients had the highest rates of meaningful improvement and the lowest rates of major deterioration. In contrast, prostate cancer patients were more prone to significant declines in QoL scales over time.

**Interpretation:**

This prospective study demonstrates that department-wide collection of PROM is feasible. Using a digital platform, we achieved a high initial response rate and successfully engaged patients across a broad range of cancers. The routine PROM collection provided valuable insights into patients’ quality of life and symptom burden during and after treatment.

## Introduction

The most important endpoints in oncology are overall survival and quality of life (QoL). The former is easy to measure and routinely reported, but although recognized as equally important, QoL is not evaluated in clinical routine. Patient-reported outcome measures (PROMs) are standardised, validated questionnaires that patients complete to measure their own functional status, symptoms, and well-being, and have proven valuable in the follow-up setting [1].

At our institution, only a small fraction (< 5%) of patients are enrolled in clinical trials at any given time, meaning that for the vast majority, detailed patient-centred outcomes are not being systematically recorded. This represents a missed opportunity to learn from real-world treatment experiences. Real-world QoL data may help to compare long-term outcomes across therapies and patient groups, guide shared decision-making, and potentially identify predictors of toxicity or areas for supportive intervention.

Barriers for implementation of QoL assessment include logistical constraints, a lack of electronic systems, and concerns about patient burden. Advances in digital health technology now enable PROM collection on a large scale with minimal disruption to clinic workflow.

In this context, we launched a program to systematically collect PROMs from all patients undergoing treatment at our department, as part of a prospective observational study. The primary aim of this analysis was to assess the feasibility of department-wide PROM collection, measured by patient participation rates at baseline and follow-up and the practicality of integrating the process into routine care. We also aim to describe preliminary findings on patient-reported QoL and symptoms over time and to explore differences between diagnostic groups. By sharing our experiences and results, we hope to inform other institutions considering large-scale PROM implementation and contribute to more patient-centred oncology care.

## Patients/material and methods

This was a prospective, non-interventional observational study conducted at the Department of Hematology, Oncology, and Radiation Physics at Skåne University Hospital in Lund, Sweden. The study period for prospective data collection was January 2022 through April 2025.

The study was approved by the Swedish Ethical Review Authority (approval number DNR 2020-04164). All patients who were invited to participate provided written informed consent before enrollment.

Inclusion criteria were age ≥18 years; referral for radiotherapy and ability to understand the study information and provide consent. Exclusion criteria were inability to complete questionnaires due to cognitive, psychiatric, or linguistic barriers. During the study period, all new patients meeting the criteria were approached, unless a treating physician judged that inclusion was not appropriate.

PROM data were collected using a custom web-based platform, Amnicare – Blå Appen (previously named Stretch Care – Blå Appen in Swedish) [2]. After obtaining written informed consent, patients received a test message with a link to securely login to access and complete the questionnaires. PROMs could be completed on the smartphone, a tablet or computer. Baseline questionnaires were completed before start of treatment. If a patient had not completed the baseline PROM by the time of treatment initiation, reminders were given to fill it out as soon as possible.

Long-term follow-up PROMs were scheduled annually, up to 5 years after treatment completion. Automatic email reminders were generated by the system at those time points for patients who remained in follow-up, and non-participants received additional reminders or a courtesy phone call if needed.

The core instrument used was the European Organisation for Research and Treatment of Cancer Quality of Life Questionnaire Core 30 (EORTC QLQ-C30) version 3.0 [3]. In addition, some diagnosis specific questionnaires from EORTC were added from February 1, 2023 (ANL27, CR29, OG25, BR23, LC13, HN35, PR25, BN20, CX24, EN24, BR22). Three questionnaires were created locally from the EORTC database to cover areas we found important. Finally, the baseline questionnaire also included a short form to capture demographic and clinical data. Clinical data were obtained from hospital electronic medical records and from national cancer quality registries.

### Statistical analysis

Feasibility endpoints were primarily descriptive. We calculated the proportion of invited patients who agreed to participate and completed the baseline PROM (baseline response rate). We also calculated follow-up response rates at 1 and 2 years post-treatment, defined as the number of patients responding at those time-points divided by the number of patients who were alive and had reached that time-point in the study. Patient characteristics were summarised for participants versus non-participants.

For analysis of PROM outcomes, we focused on key QLQ-C30 scales and examined their median and mean values at baseline and follow-up. A change of ≥ 10 points on a 0–100 scale was considered clinically significant [4]. For each diagnostic subgroup we determined the proportion of patients who experienced ≥ 10-point improvement or deterioration in select scales between baseline and follow-up. All statistical analyses were performed using R (v4.5.2) and Python (v3.11.5) software.

## Results

In total 12,742 patients were referred for radiotherapy during the study period between January 2022 and August 2025; analyses in this report include data through April 7, 2025 (data-lock). A total of 4,764 patients (37%) provided informed consent for the PROM program. An additional number of cases were excluded from the analysis due to data entry errors or incomplete key information. After these exclusions, 3,699 (77.6%) remained. Among these, 3,056 patients (82.6%) provided at least one usable PROM questionnaire at baseline and were included as participants.

The remaining patients were either unable to complete the baseline questionnaire despite consenting, due to illness, technical difficulties (no smartphone, tablet or computer), change of heart, or other reasons. The reason for not participation included poor health, cognitive dysfunction, inability to comprehend spoken or written Swedish. However, the ethics and trial design disallowed analysis of the patients and their decision to decline to participate in the trial. The median age of participants was 69 years (interquartile range 59–76), and 52% were female. By comparison, the group of patients who did not participate (*n* = 9,416 had a median age of 71 (61–78) and a slightly higher proportion of males (Table 1). More striking were the differences in diagnostic distribution. Patients with breast and prostate cancer together constituted more than half of the participants, 35% and 20% of participants, respectively, whereas they accounted for only about 23% and 17% of non-participants. This indicates that patients with these common, often less immediately life-threatening cancers were more likely to engage with the PROM program. In contrast, patients with diagnoses reflecting metastatic disease or very poor prognosis were relatively under-represented: for instance, metastatic bone involvement comprised 11.0% of non-participants but only 5.9% of participants, and metastatic cancer in brain was 4.4% of non-participants versus 2.7% of participants.

**Table 1:** Characteristics of participants versus non-participants.

Characteristic	Participants	Non-participants
*N*	3,056	9,416
**Age**	69 (IQR 59–76)	71 (IQR 61–78)
**Diagnosis**		
Breast cancer C50.9	1,083 (35.4%)	2,155 (22.9%)
Prostate cancer C61	604 (19.8%)	1,609 (17.1%)
Secondary malignant neoplasm of bone C79.5	180 (5.9%)	1,032 (11.0%)
Lung cancer C34.9	159 (5.2%)	603 (6.4%)
Rectal cancer C20	142 (4.6%)	452 (4.8%)
Secondary malignant neoplasm of brain C79.3	83 (2.7%)	411 (4.4%)
Primary malignant neoplasm of brain C71.9	56 (1.8%)	228 (2.4%)
Secondary malignant neoplasm of lymph node C77.9	53 (1.7%)	141 (1.5%)
Intraductal *in situ* breast cancer D05.1	46 (1.5%)	105 (1.1%)
Anal carcinoma C21.0	40 (1.3%)	120 (1.3%)
Tonsillary cancer C09.0	30 (1.0%)	102 (1.1%)
Malignant neoplasm of corpus uteri C54.9	28 (0.9%)	84 (0.9%)
Bladder cancer C67.9	23 (0.8%)	142 (1.5%)
Multiple myeloma C90.0	17 (0.6%)	113 (1.2%)
Malignant neoplasm of cervix uteri C53.9	16 (0.5%)	118 (1.3%)

IQR: interquartile range.

### Longitudinal response

**Figure 1:**
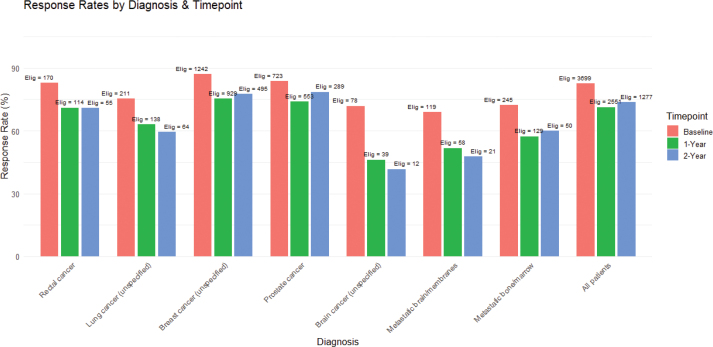
Response rate per diagnosis at baseline and 1 and 2 years after end of treatment.

Figure 1 depicts the longitudinal response rates at the specified follow-up intervals. At the baseline time-point, the response rate among those who had consented to participation was 82.6%. At 1-year, out of the patients who were alive and had reached one year post-treatment, 71.3% completed the PROM (Figure 1). By the 2-year follow-up, the response rate declined (73.7%). It is worth noting that older patients (≥ 70) were just as likely to remain in the program as younger patients. Very few patients cited technical difficulties as a reason for not participating after baseline. Instead, patients claiming technical difficulties already declined participation at baseline.

### Diagnoses and clinical characteristics of participants

Breast cancer was the single largest group, followed by prostate cancer. Patients with very poor prognosis cancers formed a smaller proportion of the long-term respondent pool. About 65% of the participants were treated with curative intent. The response rate was somewhat higher among patients in the curative treatment group compared to palliative, especially by the 1- and 2-year marks.

### Quality of life outcomes – Overall and by diagnosis

**Figure 2:**
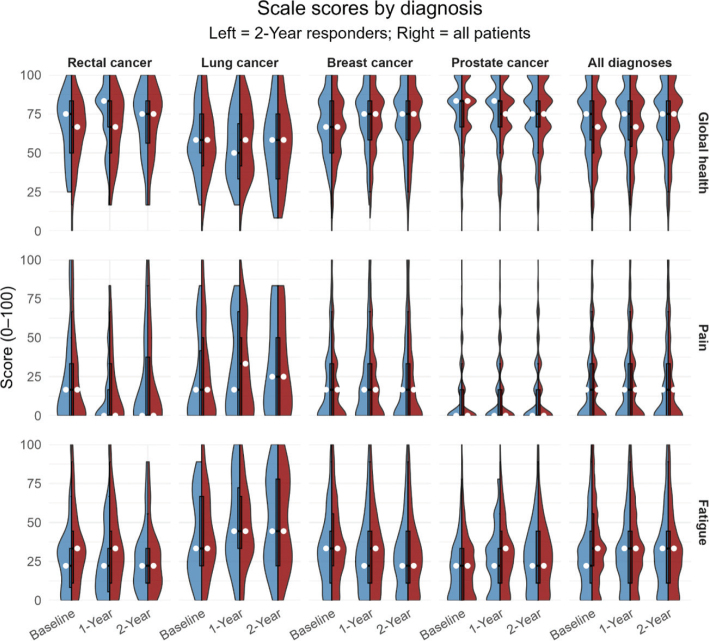
Global health, pain, and fatigue scores by diagnosis at baseline and 1 and 2 years after treatment. Split violin plots show patients who responded at 2-year follow-up (blue, left) and all patients (red, right). Boxplots indicate the interquartile range, with the median shown as a white dot.

The PROM responses enabled us to evaluate patients’ self-reported QoL across various domains. At baseline, prior to therapy, the mean Global Health Status/QoL score for all patients was 65, although this varied by diagnosis: baseline global QoL was highest in patients with prostate cancer (75) and breast cancer (66), and lowest in patients with lung cancer (58). Figure 2 (right panel) illustrates some of these baseline differences in global QoL, fatigue, and pain scores across diagnoses. Prostate cancer patients had relatively favourable scores – higher global QoL and lower symptom scores for fatigue and pain at baseline – whereas lung cancer patients reported higher fatigue and lower global QoL on average at baseline.

By one year after treatment, global QoL scores showed a slight improvement or remained stable in many groups. The mean global QoL for all patients who completed the 1-year follow-up was 68, a small increase from baseline. Notably, prostate cancer patients experienced worsening fatigue and reduced QoL at 1 year.

At 2 years, the trends observed at 1 year largely persisted among those still reporting. Many breast cancer survivors continued to report excellent or improved QoL. In contrast, prostate cancer patients as a group showed a sustained reduction in global QoL at 2 years relative to baseline, along with elevated fatigue levels.

### Cross group comparisons

To better visualise differences between diagnoses, we focused on three representative scales – global health status, fatigue and pain (Figure 2). Among 2-year participants, the average baseline global health score was higher or equal in each diagnostic category compared to the baseline average of all patients in that category.

### Clinically significant changes in QoL

**Figure 3:**
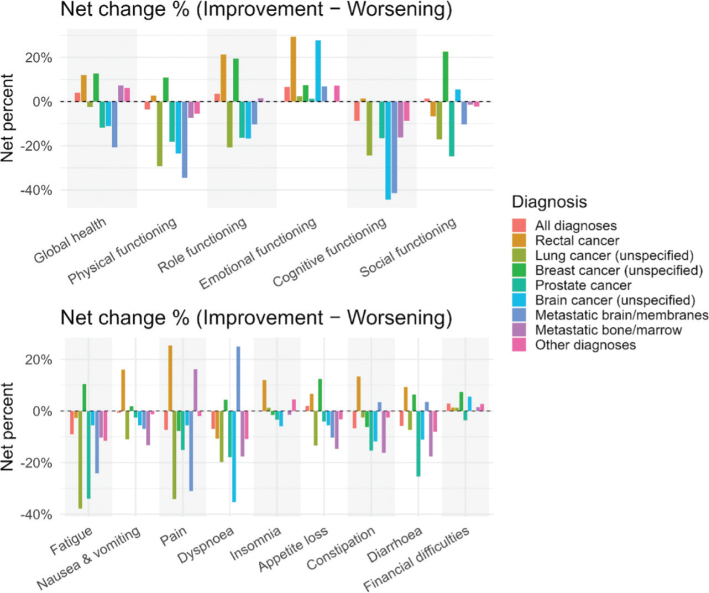
Net change by diagnosis between baseline and 1 year follow up for all EORTC-QLQ C30 scales.

We evaluated the proportion of patients with clinically significant changes (improvement or deterioration of ≥ 10 points) in QoL scales from baseline to follow-up. Among breast cancer patients, 31% reported a ≥ 10-point improvement in global health by one year, and relatively few (< 20%) showed a significant decline. They also had high rates of improvement in fatigue compared to other diagnosis. On the contrary, prostate cancer patients showed a different pattern: a considerable net fraction (30%) had a ≥ 10-point deterioration in fatigue (Figure 3). Patients with colorectal cancer showed improvements in both functional and symptomatic scales.

Notably, patients with advanced or metastatic disease showed high rates of deterioration and low rates of improvement. A majority of patients with metastatic bone disease who were still alive at one year reported worse fatigue scores over time, which is consistent with disease progression.

### Feasibility outcomes

From an implementation standpoint, the study demonstrated feasibility in several dimensions:

Sustainability: The gradual decline in response rates to 73.7% by two years, while notable, is within expected ranges for longitudinal studies. Importantly, a majority of patients remained in the program at long-term follow-up, indicating that the burden of annual PROM surveys was acceptable.

Integration into Workflow: The PROM collection process was successfully integrated into clinic operations with minimal disruption. Nurses managed enrolment and reminders as part of routine visits. After some initial adjustments, the rate of missing data due to technical issues became very low.

Data Quality: The completeness of questionnaires was high – most returned forms had very few missing answers. We did exclude some cases due to inconsistencies, but these were uncommon. For EORTC QLQ-C30, internal consistency of multi-item scales in our cohort was high (Cronbach’s alpha > 0.8 for all functional scales).

## Discussion and conclusions

In this study, we demonstrated the feasibility of implementing a department-wide PROM collection program in a radiotherapy and oncology department, covering thousands of patients across diverse cancer diagnoses. To our knowledge, this represents one of the largest real-world evaluations of routine PROM collection in a single cancer centre. Our experience is in line with reports from ePRO implementations from other centres, which have found strong patient acceptance and even improved outcomes when PROMs are used routinely [5–8]. We found that once patients were enrolled, retention at one year was robust, though by two years there was moderate attrition. The retention rate of >70% at two years, while leaving room for improvement, compares favourably with prior studies in routine practice, which have documented retention around 50–70% at 1 year [5]. Importantly, much of our attrition was due to clinical reasons rather than patients actively opting out; relatively few patients withdrew consent or refused further surveys while still under follow-up.

One key challenge revealed by our results is selection bias among PROM respondents. We observed that patients with more severe or advanced diseases were less likely to enter or remain in the PROM program. This led to under-representation of certain groups (e.g. those with metastatic cancer) in the longitudinal data. Consequently, the QoL outcomes at later time points may paint a somewhat optimistic picture because the sickest patients are missing. We have attempted to address the recruitment of the sickest patients in other related endeavours (beyond the scope of the current work).

Our analysis of QoL trajectories by cancer type yielded insights consistent with clinical expectations and literature. Breast cancer patients had generally good and improving QoL after treatment, which matches prior research showing high recovery and adaptation in early-stage breast cancer survivors [9–12]. Prostate cancer patients, on the other hand, often experience chronic side effects, especially from androgen deprivation, that can impair vitality and function; our finding of sustained or worsened fatigue in this group is in line with known impacts of hormonal therapy on long-term QoL [13, 14]. Lung cancer patients’ low baseline QoL and further deterioration for those who survive reflect the serious symptom burden associated with lung malignancies, as documented in other studies [15, 16].

From a clinical implementation perspective, one encouraging observation is that routine PROM collection can be integrated without disrupting standard care. Our staff managed to embed the process of PROM enrollment and follow-up reminders into their workflow relatively smoothly, facilitated by the use of automation. The feasibility is further supported by the fact that very few participants cited difficulty with the technology – an important consideration in an older population.

One of the goals of collecting PROMs systematically is to use the data to improve patient care in real time. Although our study’s primary focus was feasibility and analysis of aggregated data, the infrastructure set up here may potentially benefit clinical management. For instance, nurses reviewing PROM responses before follow-up appointments could identify patients reporting severe symptoms and flag them for intervention. Indeed, evidence suggests that incorporating PROM monitoring into routine care can reduce emergency visits and improve outcomes by catching issues early [5]. Experience from Canada also highlights that if PROM are collected, they should be used actively by clinicians, otherwise the incentive for patients to continue reporting will be diminished [17]. Possibly, more active engagement of patients and caregivers in the development of PROM tools may improve their clinical implementation [18].

Our study has several strengths: a large sample size covering virtually all tumor types, high response rates, and use of well-validated instruments (EORTC QLQ-C30 and PRO-CTCAE). It provides a real-world benchmark for what proportion of patients can be engaged in routine PROM collection at a busy cancer centre. We also report granular data on differences between participants and non-participants, which is rarely described in feasibility studies. The use of a structured, electronic data capture with integration to clinical data is another strength, enhancing data completeness and quality.

We also acknowledge some limitations. Firstly, this is a single-centre experience in a Swedish university hospital, which may limit generalisability. Our patient population is relatively homogeneous, mostly Caucasian, with universal healthcare access, and results may differ in other health systems or cultural contexts. One limitation of the use of PROM is that patients need to be able to respond independent of an interpreter or family member, limiting its use in patients of foreign descent and in patients with cognitive impairment. Secondly, as mentioned, there is an inherent bias in the data due to patient drop-out; we did not impute data for those who did not respond due to protocol, so our longitudinal findings pertain to completers only. Thirdly, we focused on feasibility and descriptive outcomes rather than testing specific hypotheses about QoL differences. Fourthly, while we included the EORTC diagnosis specific questionnaires, in this report we did not separately analyse those symptom-specific data in detail, which will be the subject of a subsequent article. Fifthly, our outcome data are limited to patient-reported metrics; correlation with clinical outcomes such as survival or tumor control was beyond the scope of this article. Finally, patients needed to understand Swedish in order to complete questionnaires, which constitutes a fraction of our patients. As per protocol, we did not record the reason for or for not reporting the PROMs data, which might have provided insight in the patterns related to enrolment (or lack thereof) in the data collection.

The successful collection of PROMs on a department-wide scale means that we now possess a rich dataset of patient-centred outcomes. This has multiple potential applications: benchmarking and identifying areas for improvement; informing patients during consultations; and supporting research such as dose–response analyses for toxicity. In fact, one of the motivations of this project was to eventually correlate PROMs with treatment details such as radiation dose distributions to better understand dose–toxicity relationships.

In summary, our study confirms that large-scale routine PROM collection is achievable and yields meaningful data. The data gathered not only benefit research and quality improvement but, most importantly, can directly enhance patient care by focusing attention on outcomes that matter to patients.

## Data Availability

The dataset includes sensitive personal information as well as data on date of diagnosis, which may be used to identify the individual, why data cannot be made open.
